# A Gesture Recognition Algorithm for Hand-Assisted Laparoscopic Surgery

**DOI:** 10.3390/s19235182

**Published:** 2019-11-26

**Authors:** Carmen López-Casado, Enrique Bauzano, Irene Rivas-Blanco, Carlos J. Pérez-del-Pulgar, Víctor F. Muñoz

**Affiliations:** Department of Systems Engineering and Automation, Universidad de Málaga, Andalucía Tech, 29071 Málaga, Spain; mclopezc@uma.es (C.L.-C.); ebauzano@uma.es (E.B.); irivas@uma.es (I.R.-B.); vfmm@uma.es (V.F.M.)

**Keywords:** surgical robotics, machine learning, gesture recognition

## Abstract

Minimally invasive surgery (MIS) techniques are growing in quantity and complexity to cover a wider range of interventions. More specifically, hand-assisted laparoscopic surgery (HALS) involves the use of one surgeon’s hand inside the patient whereas the other one manages a single laparoscopic tool. In this scenario, those surgical procedures performed with an additional tool require the aid of an assistant. Furthermore, in the case of a human–robot assistant pairing a fluid communication is mandatory. This human–machine interaction must combine both explicit orders and implicit information from the surgical gestures. In this context, this paper focuses on the development of a hand gesture recognition system for HALS. The recognition is based on a hidden Markov model (HMM) algorithm with an improved automated training step, which can also learn during the online surgical procedure by means of a reinforcement learning process.

## 1. Introduction

Minimally invasive surgery (MIS) has become one of the most important surgical techniques, because of its capability of reducing the postoperative convalescence for patients. However, MIS imposes several restrictions to the surgeon’s perceptions and freedom of movement, such as the loss of visual depth perception, limited tactile sensation, and the reversed movement of the laparoscopic tools.

MIS techniques have been evolving over time to achieve two opposite goals, either for the sake of the patient or to reduce the surgeon’s restrictions as mentioned above. Single-incision laparoscopic surgery (SILS) is an example of an MIS variant within the first goal. This technique requires only one incision into the patient, though the management of the surgical tools is seriously limited. On the opposite side, hand-assisted laparoscopic surgery (HALS) consists of introducing one of the surgeon’s hands into the surgical workspace to improve tactile sensation and dexterity, but the incision required to introduce the hand is longer than the ones for the traditional laparoscopic tools used in other MIS techniques.

The introduction of robotic assistants as co-workers into MIS techniques [1] aims to improve both the patient’s outcomes and the surgeon’s movements and perceptions. This kind of robotic system must have an intuitive human–machine interface (HMI) so it can assist the surgeon properly during the surgical procedure. Simple buttons or direct commands (i.e., voice) are not fast enough to make a fluid communication with a surgical robot, and lead to interruptions during the surgery. However, the HMI proposed in [2] for controlling the endoscope through the surgeon’s eye movement or the one presented in [3], where surgeons’ multimodal communication cues are analyzed and used to perform turn-taking prediction with a robotic scrub nurse, are examples of appropriate HMI. One relevant research field of robotic assistant HMI systems focuses on the recognition of the surgeon’s tasks. This recognition is usually based on the detection of tool gestures [4], already studied in our previous works [5], or hand gestures if HALS is used. From the tool gesture point of view, there is relevant work for benchmarking purposes that involves both the publication of general datasets with the DaVinci robot system and the classification of the main recognition methodologies [6]. On the other hand, there are examples of hand gesture recognition in surgery, such as the ones to command a robotic nurse to assist surgeons by passing surgical instruments [7,8,9]. However, the recognition of hand gestures inside the patient has not been studied in such depth. The main problem of HALS lies in the limited space available to move the hand inside the abdominal cavity, as opposed to the usual application of hand gesture recognition methodologies, which consider relevant displacements of the hand [10]. This is one of the main reasonswhy the surgeon’s hand anatomy must be mapped before the recognition technique itself. The mapping methodologies can be classified into two main categories: depth-map-based methods (volumetric features) and skeleton-based methods (joint and angle features) [11]. Although digital cameras are the most common sources of data extraction, other works prefer the use of three-axis accelerometers [12].

The hands are the most relevant anatomic area to detect the surgeon’s gestures. The literature presents several algorithms that are used for hand gesture recognition (HGR) [13], such as hidden Markov models (HMMs), conditional random field (CRF), or pure signal comparison with dynamic time warping (DTW). The first ones are used in some works in applications such as automatic sign language recognition through an accelerometer glove [14] or electromyographic signal pattern recognition [15]. Variations of the CRF method like Markov/semi-Markov CRF are obtaining good results for surgical gesture segmentation and classification with the combination of video and kinematic data [16]. On the other hand, the latter (DTW) has been used in the real-time recognition of hand gestures [17] or the detection of behavioral patterns of attention deficit hyperactivity disorder [18]. Each algorithm has its own advantages and drawbacks [19], but their recognition success rates are very similar. These gesture recognition algorithms require metrics and clustering methods to compare and classify new measured data with reference patterns. The detection of thresholds for comparative analysis are commonly based on Euclidean, Hamming [12], or most recently, Mahalanobis distances [20]. Moreover, the clustering classification is handled by means of techniques like the k-means [21] or the Gaussian mixture model (GMM) [22].

This paper proposes an enhanced HGR algorithm based on HMM techniques and a reinforcement learning process to be used in a HALS procedure. The core of this algorithm is the automation of the HMM training, coupled with an on-line update of the gesture library, considering the confidence index of the recognized gesture. The main reason for using a generative learning method like the HMM instead of a discriminative model like CRF is its faster convergence to asymptotic error [23]. Although this method may retain a slightly higher error than discriminative models, this drawback can be handled by a reinforcement learning (RL) algorithm [24] that updates the HMM gesture library online.

## 2. Materials and Methods

### 2.1. Need for Gesture Recognition in HALS

**Figure 1:**
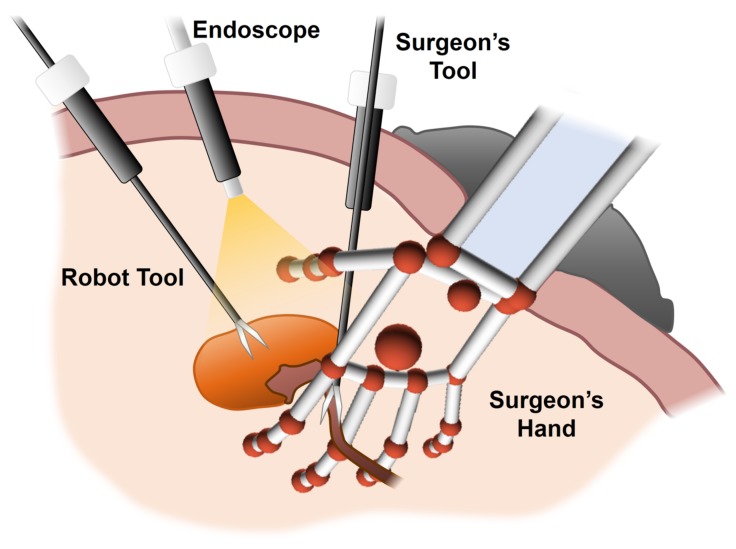
The hand-assisted laparoscopic surgery (HALS) workspace is composed of: a tool and an endoscope handled by a two-arm surgical robot; another tool managed by the surgeon; and finally, the surgeon’s hand. All these components are inside the abdominal cavity.

The HALS technique requires the presence of one surgeon’s hand within the surgical workspace while the other one manages the laparoscopic surgeon’s tool, as shown in Figure 1. This setup does not allow the use of an additional tool, which is usually helpful for several standard laparoscopic procedures. To minimize this restriction, previous work [5] proposes the use of a two-arm surgical robot assistant. One arm orientates the endoscope towards the region of interest, and the other one handles an extra robot tool. This second tool allows laparoscopic procedures that require both tools (e.g., suturing) to be performed by means of automatic collaborative movements.

The actions of the robot assistant primarily depend on the ongoing task of the surgeon. Movements made by the surgeon’s hand are different depending on the stage of the surgical procedure. Thus, the main goal consists of rendering these movements as gestures that are understandable by the robot assistant. These gestures can be either surgical tasks or direct orders for the robot assistant (e.g., hold a tissue or point with finger to focus the camera at a specific position). In both situations, there are several methods to proceed with every surgical task (i.e., suturing, knot tying, etc.), thus each surgeon performs the intervention in a unique manner [25]. Besides, the morphology of each surgeon’s hand (i.e., size and length of the phalanges) may lead to different trajectories of the fingers for the same task. For these reasons, the recognition system must be trained for each surgeon. Each surgeon will have an associated gesture library where the specific gestures to perform in the surgery will be stored.

### 2.2. Cognitive Architecture for HALS Protocols

**Figure 2:**
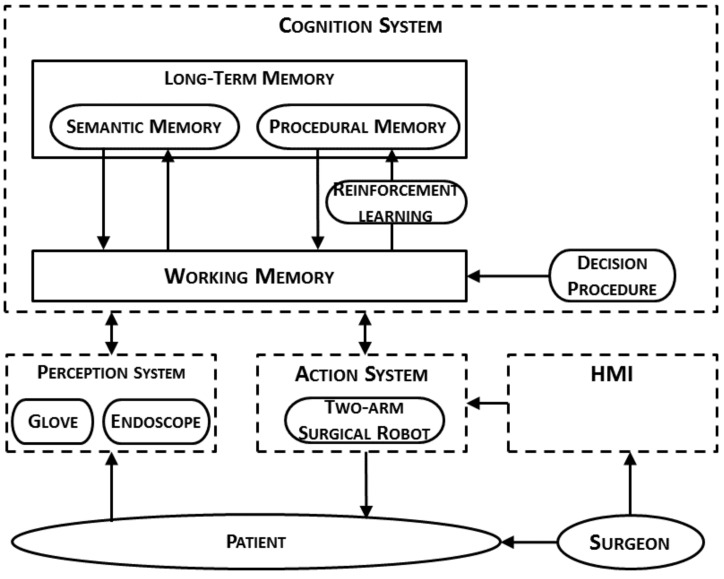
Cognitive robotic architecture for HALS protocols. HMI: human–machine interface.

The architecture proposed for HALS protocols follows a cognitive approach (Figure 2). It is divided into three main systems: perception, cognition, and action. The perception system measures data from the surgical workspace with the aid of two sensors. Firstly, the image analysis of the endoscope camera allows tracking of the tools or any other relevant object in the abdominal cavity. This information is then used by the action system to plan a trajectory for the autonomous tools. Secondly, a smart glove acquires information about the hand pose and orientation, which describes the gesture of the current surgical stage. This information is the input of the HGR algorithm proposed in this paper.

The cognition system is based on production rules to govern the behavior of the architecture. These production rules are supported by a space where the long-term memory information is combined with the working memory. The former encodes the knowledge base acquired through the analysis of long periods of time; the latter includes information about what is currently happening, introducing perception data and the actions to be performed. The cognition system also includes learning algorithms—specifically, reinforcement learning algorithms—to enhance the performance of the architecture.

Finally, the *action system* oversees execution of the decisions made by the *cognition system*. These decisions are related to the surgical robot movements to suitably assists the surgeon in each surgical stage. Although the surgical robot is intended to act autonomously, the surgeon may override these movements (or even command an emergency stop) by means of an HMI system.

**Figure 3:**
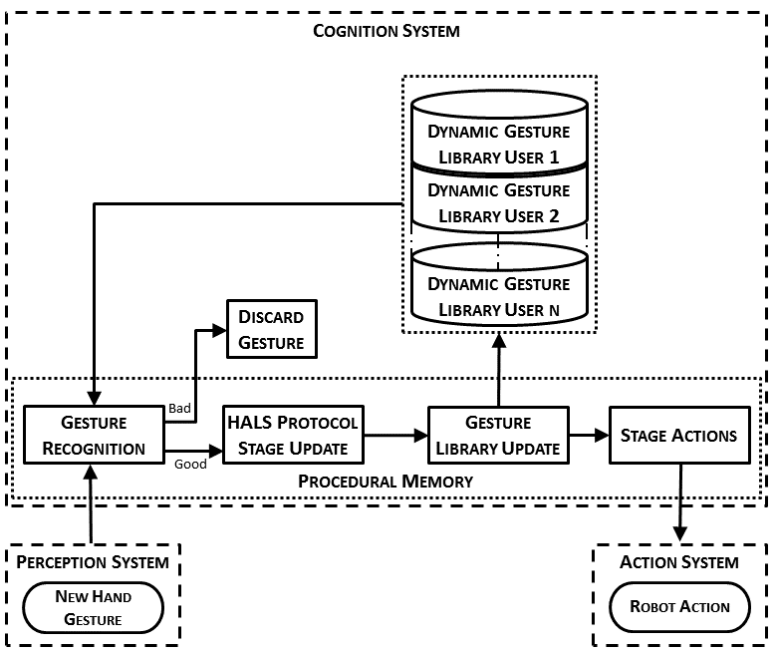
General system workflow for a HALS protocol showing both the standard path when the gesture is well detected, and the error path when it is misrecognized.

The way a HALS protocol is included into the cognitive architecture is outlined in Figure 3. This figure shows the general workflow of a protocol for HALS including the hand gesture recognition and the main interactions between the different systems in the architecture. When the perception system detects that a new hand gesture has been made, the gesture recognition algorithm tries to recognize it. The recognition of this gesture makes the HALS protocol evolve from one stage to another. Then, the dynamic gesture library is modified with the information of the newly recognized gesture. Finally, depending on the HALS protocol stage, the actions to be performed by the surgical robot to properly assist the surgeon are communicated to and then executed by the action system.

The different robot actions have been further analyzed and verified in previous works of our group. The endoscope motion combines a reactive behavior based on instrument tracking with a proactive behavior based on the surgery workflow [26]. This control makes it possible to accommodate the camera view to the current state of the task with enough flexibility enable it to adapt its behavior to unplanned or unforeseen situations.

Regarding the tool movement managed by the robotic assistant, a hybrid force–position controller is implemented based on [5]. On one hand, the position controller navigates the robot tool, and consists of an artificial potential fields algorithm modified to adapt the velocity depending on the approach to an obstacle (i.e., the surgeon’s hand/tool). On the other hand, the force controller is designed to apply a force with the tool by means of a Proportional-Integrative (PI) controller feedback.

The normal workflow can be altered in case of a bad result of the gesture recognition algorithm. This result may be because of an intentional gesture that was poorly recognized, or an unintentional movement of the hand made by the surgeon, which eventually is considered as a gesture by the recognition algorithm. In both situations, the recognized gesture is labeled as a bad result if the gesture does not correspond to a valid action in the current stage of the surgical protocol. Moreover, if the gesture is the one expected but its confidence index is below a threshold, the system also labels it as bad. When a gesture is labeled as bad, the system will send a voice message to the surgeon. Otherwise, the surgeon may still use a voice command interface to correct an unexpected detection of the recognition system, making use of the voice decoder included in the architecture presented in our previous work [5]. This command must be explicit enough to avoid confusion when the surgeon talks with the surgical human staff. This action leads to a correction on the detected gesture, which prevents an undesired update of the dynamic gesture library.

#### 2.2.1. Semantic Memory

The semantic memory coupled with the procedural memory form part of the long-term memory of the cognition system. This memory includes concepts, meanings, facts and any other forms of knowledge needed to understand the environment [27]. More specifically, the knowledge needed to suitably work in a HALS workspace includes (1) the different surgical protocols that the surgical robot is able to assist; (2) the sequence of stages for each of those protocols, and (3) the actions executed in each stage. All the information listed above is modeled as a database with tables or semantic units, $S_{i}$ (i∈1,2,3), that store the knowledge needed to properly assist the surgeon. Each unit entry includes a set of attributes to suitably define it. The first semantic unit S1 lists all the HALS protocols where the robotic assistant is going to be used. Each entry of this unit consists of two attributes: the protocol name (*protocolName*) and the protocol identifier ($protocolId_{j}$). Hence, $S_{1}$ is defined as:

(1) $$\begin{array}{c} S_{1}=\left\{S_{11},S_{12},S_{13}\cdots \right\},\\ S_{1j}=<protocolId_{j},protocolName_{j}>. \end{array}$$

The second semantic unit $S_{2}$ describes the different stages of each protocol and all the trigger signals (i.e., the events that make the protocol progress from one stage to another). Each stage stored into $S_{2}$ is defined by three attributes: the protocol identifier ($protocolId_{j}$), the stage ($stage_{j}$), and the trigger ($trigger_{j}$). Hence, each entry of $S_{2}$ is defined as:

(2) $$S_{2j}=<protocolId_{j},stage_{j},trigger_{j}>.$$

For each stage, the semantic unit $S_{3}$ stores the corresponding actions that the surgical robot must make to assist the surgeon. Therefore, for each stage there will be as many entries as actions to be executed. More specifically, the HALS clipping protocol presents two different autonomous movements: one for the endoscope, and another for the robot tool. Hence, each entry of $S_{3}$ is defined as:

(3) $$S_{3j}=<protocolId_{j},stage_{j},action>,$$

where *action* is the action to be performed for each surgical element, endoscope, and/or surgical tool.

#### 2.2.2. Procedural Memory

The procedural memory is the long-term memory that manages the knowledge of when, how, and what actions have to be executed by connecting internal and external data. The internal data come from the semantic memory, while the external data are provided by the perception and action systems. This knowledge is presented as IF–THEN rules, the so-called production rules, where each production has a set of conditions and a set of actions. The conditions denote the “IF” part of the rule and check the current state of the task, whereas the “THEN” part defines the actions to be executed.

#### 2.2.3. Working Memory

The working memory is the short-term memory of the architecture where the current environment situation is codified. It includes not only the current cognition system situation, but also that of the rest of the architecture (i.e., the perception and action systems). All this information is used by the decision procedure to infer which production rules in the procedure memory must be applied next.

### 2.3. Offline Gesture Training Process

The first element of the general system workflow introduced in Figure 3 consists of the gesture recognition. This algorithm requires the initial training of a dynamic gesture library, which is unique for each surgeon as previously described in Section 2.1. Thus, this section explains the data acquisition and processing steps followed in the present paper to obtain the required model for each gesture within the dynamic gesture library.

The gesture training process has been designed in such a way that only the most relevant information of a gesture is automatically processed and encoded into sequences of tags, as described in Section 2.3.1. Such data are then processed by the gesture training algorithm (see Section 2.3.2), which is implemented by means of an HMM network, to obtain a set of patterns for each of the proposed gestures.

#### 2.3.1. Data Acquisition and Processing

**Figure 4:**
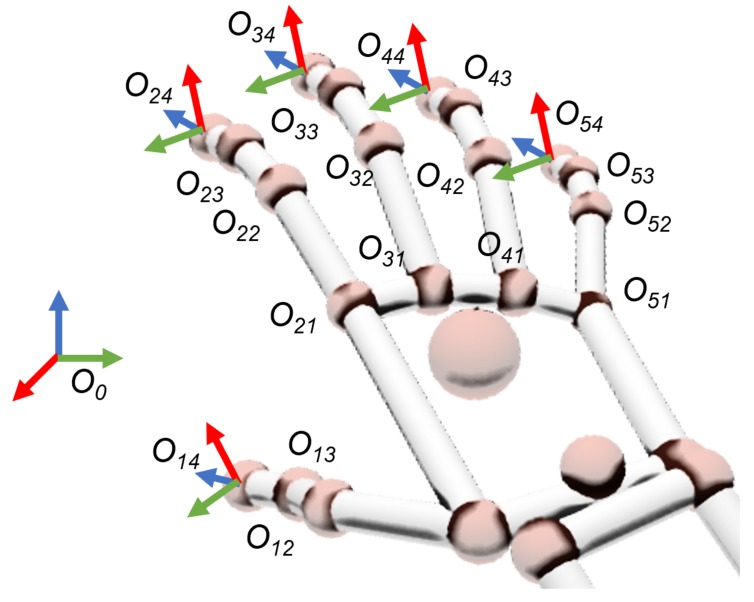
Coordinate frames related to all finger joints and tips. Green, red and blue arrows represent the XYZ axis respectively.

The gesture training process is performed on a patient simulator before the intervention, and begins with the data acquisition of the surgeon’s hand movements by means of a smart glove. This sensor device measures the origin of the coordinate frames $O_{ij}$ for each of the joints of the surgeon’s fingers relative to the reference frame of the sensor device $O_{0}$ (Figure 4). On each frame, index *i* denotes the finger element (1 = thumb; 2 = forefinger; 3 = middle; 4 = ring; 5 = pinky) and *j* refers to the joints between their contiguous bones (1 = metacarpal–proximal; 2 = proximal–middle; 3 = middle–distal; 4 = fingertip). All coordinate frames $O_{ij}$ are oriented in such a way that their $z_{ij}$ vectors have the direction of the *j* phalanx related to finger *i*. These frames, defined by their homogeneous transform matrices $T_{ij}^{0}$ related to frame $O_{0}$, can be redefined as a set of geometrical parameters (so-called features) like the joints’ flexion $q_{ij}$, the distance $l_{i}$, and the orientation $\beta_{i}$ between contiguous fingertips:

(4) $$\begin{array}{c} l_{i}=\;\left\|P_{(i+1)4}^{i4}\right\|,\\ \beta_{i}=atan2(\|z_{i4}^{0}z_{(i+1)4}^{0}\left\|,z_{i4}^{0}z_{i+14}^{0}\right),\\ q_{ij}=atan2(\|z_{\mathrm{ij}}^{0}z_{i(j+1)}^{0}\left\|,z_{\mathrm{ij}}^{0}z_{\mathrm{ij}+1}^{0}\right),\\ f_{g}=\langle l_{1}\cdots l_{4}\;\beta_{1}\cdots \beta_{4}\;q_{12}\cdots q_{53}\rangle =\langle f_{x}\rangle , \end{array}$$

where $P_{i4}^{(i+1)4}$ is the distance vector among two contiguous fingertips.

**Figure 5:**
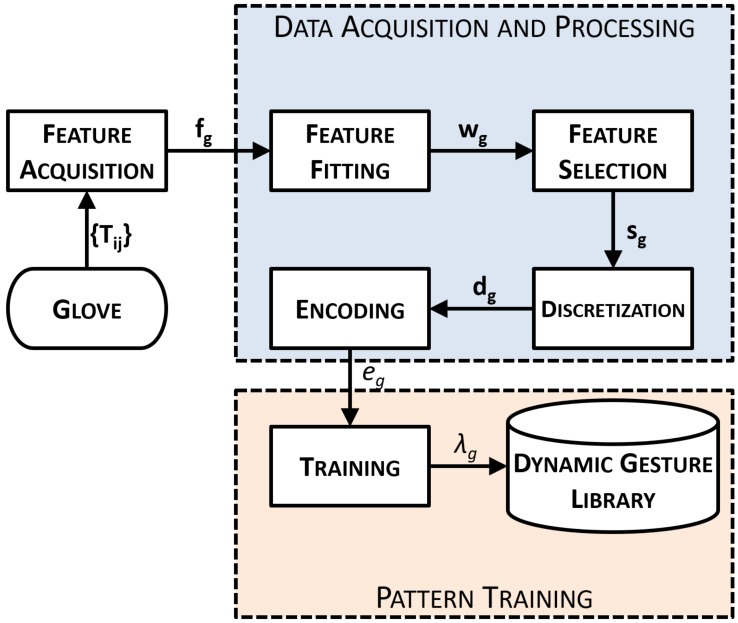
The gesture training process workflow divided into data acquisition processing and pattern training.

In (4), all features for a specific gesture *g* are gathered as a component $f_{x}$ into a feature vector $f_{g}$. This new definition of the full hand pose in terms of features simplifies the training process workflow, which appears in Figure 5. However, this set of 22 features cannot be used together on the HMM training process because of the exponential growth of their combinations into possible states of the hand. Thus, one major contribution of this work consists of an algorithm which selects the features that best represent each trained hand gesture, discarding the rest of them. The main advantage is its capability of finding the most representative features automatically, regardless of the trained gesture.

As a first step of the process workflow, the feature acquisition element records *N* times a specific gesture *g*. Each repetition *n* stores a feature sequence with the full trajectory of all the feature vectors $f_{g,n}\left[k\right]$ discretized with $K_{n}$ samples in time intervals of Δt. Hence, the set of all feature sequences $F_{g}$ is (5):

(5) $$F_{g}=\left\{f_{g},n\left[k\right],n\in 1\cdots N,k\in 0\cdots K_{n}\right\};t_{k}=k\Delta t.$$

Both the trajectory and velocity of the hand may change for each repetition *n*. Thus, the number of $K_{n}$ samples acquired can also be different. The segmentation condition to start and finish the recording of a feature sequence $F_{g}$ is given in terms of the energy vector $B_{g,n}$ per second of the feature vector $f_{g,n}$. More specifically, let τ be the frequency of $f_{g,n}$ (number of samples per second). The expression of the energy is (6):

(6) $$B_{g,n}={\langle b_{x}\rangle }_{g,n}=1/\tau \sum_{k=0}^{\tau -1}{\langle f_{x}^{2}\rangle }_{g,n}\left[k\right].$$

If the energy $b_{x}$ of any feature component exceeds a threshold $\epsilon_{x}$, then the feature acquisition starts recording a new gesture. Each threshold $\epsilon_{x}$ can be automatically determined with a previous experiment, where the sensor reads the features of the hand without any movement of the fingers. In case of a very long sequence of gestures, the reliability of the HMM network may drop significantly. To prevent this issue, a maximum limit for the time frame is imposed.

**Figure 6:**
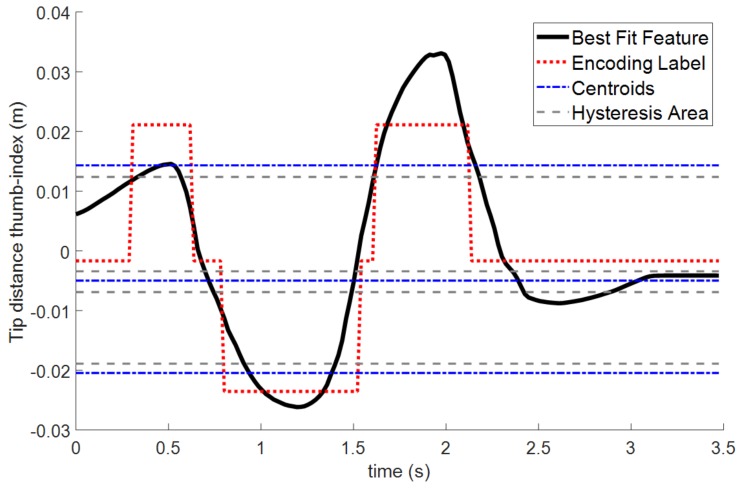
Encoded sequence and centroid values for a specific feature, including the hysteresis areas of non-actuation.

With the acquisition of $F_{g}$, these raw data must be processed in the following steps of the workflow shown in Figure 5 to eventually obtain the encoded sequence for an HMM Network:

1. Feature Fitting. Each feature sequence $f_{g,n}$ of all *N* repetitions must be comparable in sampling, time, and size. The dynamic time warping (DTW) algorithm can be used to find an optimal alignment between such feature sequences [28]. More specifically, DTW corrects the delay, equals the number of samples $K_{n}=K$, and provides a quantitative value of the similarity $\sigma_{nm}$ among two different feature sequences $f_{g,i}$ and $f_{g,i}$, being i,j∈1⋯N. In this way, the closer $\sigma_{nm}$ is to 0, the more similar are the two sequences. DTW is applied in this step to remove any repetition, which is labeled as dissimilar because of errors in sensor measurements. This process eventually leads to a reduced amount of M<N valid set of feature sequences $W_{g}=DTW\left(F_{g}\right)$ with fixed length of *K* samples.
2. Feature Selection. The training of a specific gesture usually requires only a feature subset $S_{g}\subseteq W_{g}$ that precisely defines the meaning of the surgeon’s hand movements. For example, a *scissors* gesture is performed by the separation of the index and middle fingertips, so most relevant features are $l_{2}$, $\beta_{2}$. In this way, a feature selection method is implemented to automatically select these most relevant features based on the following criteria:
  - Similarity. For each feature component $f_{x}$, the DTW is applied with respect to the other features of vector $f_{g}$. If two or more components $f_{x}$ are similar, then only one is used for training the gesture. As before, this comparison is made in terms of the Euclidean distance $\sigma_{nm}$ given by the DTW algorithm.
  - Relevance. Feature sequences with maximum peaks under a specific threshold are ignored. In other words, features with non-relevant motion are ignored for the training. These relevance thresholds are proportional to the energy thresholds obtained on the equation explained in (6). The usual values of such thresholds are about 1 cm for distance features $l_{i}$ and $15^{\circ }$ for angle features $\beta_{i}$, $q_{ij}$. Such thresholds are considered with the HALS restriction of finger movements in mind.
3. Discretization. The selected feature sequences, $S_{g}$, are processed by a discretization method, which finds the optimal amount of clusters, $c_{x}$, and stores their locations into a vector of center values, $C_{g}=\left(c_{1},c_{2}\cdots ,c_{x}\right)$. The algorithm chosen for this task is the X-means [29], a variant of the K-means that computes the optimal number of clusters by means of a specific criterion like the Calinski–Harabasz evaluation [30]. The main advantage for using X-means lies in the automation of the centroids selection process. These center values $C_{g}$ are used for discretizing the selected features $S_{g}$ into $d_{g}$ by means of the Euclidean distance of each feature sample with each component center value (see Figure 6). Besides, a hysteresis zone near each center value is considered to avoid peak values and minimize unstable oscillations because of noise effects. Final values on each discretized feature component $d_{x}\left[k\right]$ are integers among 0 and its number of center values ($d_{x}\left[k\right]\in 0\cdots dim(C_{g})-1$).
4. Encoding. All the discretized sequences, $d_{g}$, of each feature component are combined into a single encoded sequence of tags, $E_{g}$, by the encoding method. The tagged sequence, $e_{g,n}\left[k\right]$, is encoded by means of the discretized feature components, $d_{x}\left[k\right]$, and the components $c_{l}$ of the center values $C_{g}$ (7):
  (7) $$e_{g,n}\left[k\right]=1+d_{1}\left[k\right]+\sum_{m=2}^{x}\left(\prod_{l=1}^{m-1}c_{l}\right)d_{m}\left[k\right].$$

This expression assigns a unique tag for each possible combination of all the discretized features.

In essence, all the steps of the data acquisition and processing were designed to determine the parameters automatically and customized for the user who is training the system. Indeed, the only parameters to be set are the energy thresholds and the relevance thresholds, which are automatically obtained by means of a prior experiment. On the other hand, the vector of center values is also automatically obtained with the X-means algorithm. Therefore, this methodology enhances the adaptation of the algorithm to every single user and their unique way of making hand gestures.

#### 2.3.2. Pattern Training

The encoded set, $E_{g}$, obtained from each repetition of the discretized feature sequences, $d_{g}$, is sequentially sent to the training process step (Figure 5). The training process constructs an HMM network associated to each of the *g* gestures explained in Section 2 by means of the Baum–Welch algorithm [31]. Each of these networks consists of a gesture set, $\lambda_{g}$, with the following trained parameters (8):

(8) $$\lambda_{g}=\left\{SQ,\;SE,\;U,\;V,\;\pi \right\}.$$

In this expression, *SQ* denotes the states of the HMM; *SE* is the set of all possible combinations of the encoded sequences, $E_{g}$, for each sample, $e_{g,n}\left[k\right]$; the transition matrix, **U**, is the probability distribution, which indicates the relations between the states; the emission matrix, **V**, is the probability distribution, which establishes the most probable value of the encoded sequence at each state; and π is the initial states distribution. More specifically, this work chose five states *SQ* for all the $\lambda_{g}$ trains. The set of encoded sequences, *SE*, includes all the combinations of $e_{g,n}$ for each sample *k*. 5×5 diagonal symmetric matrix where the upper and lower diagonals are not null, and the sum by rows (columns) is equal to 1. Likewise, **V** is initially a $5\times (c_{1},c_{2},\cdots ,c_{x})$ matrix, where the sum by row is equal to 1.

After all the *N* repetitions performed for each gesture *g*, the resulting gesture sets, $\lambda_{g}$, are stored in the dynamic gesture library. These data are used by the gesture recognition process, which is explained in detail in the next section.

### 2.4. Gesture Recognition Process and Dynamic Gesture Update

**Figure 7:**
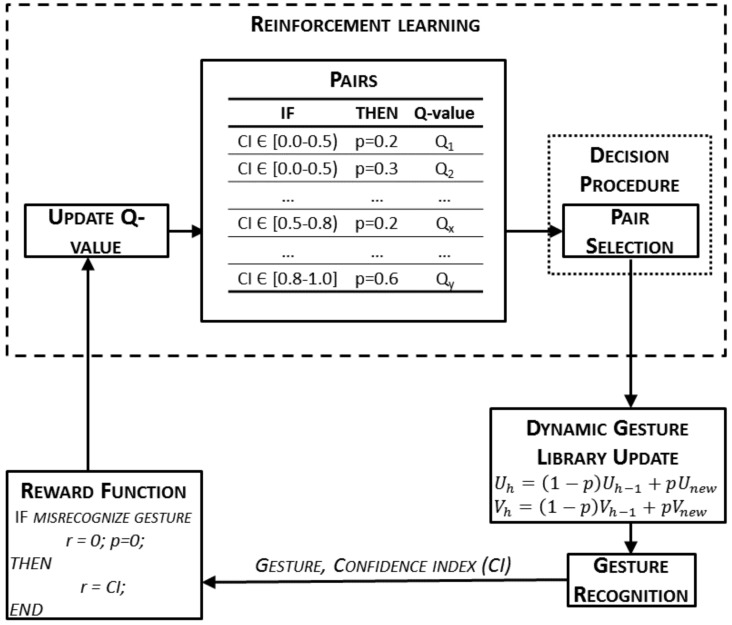
On-line learning process workflow. Update of the dynamic gesture library through the reinforcement learning (RL) process.

The dynamic gesture library that is used during the recognition process is composed of the gesture records that were made during the off-line training process. In order to include new records of the gestures already trained but made during the surgery itself, an on-line procedure to include them was designed. This way, the dynamic gesture library can adapt itself to the smooth changes the surgeon could make in the trained gestures and consequently, to improve the overall performance of the recognition process. In the following, the whole process from the gesture recognition to the update of the library will be detailed (Figure 7).

Once a new record has been recorded and encoded, the recognition process starts. The on-line encoded sequence, *E*, is processed by a forward–backward algorithm for each gesture set, $\lambda_{g}$, of trained data. This algorithm computes the probabilities, $P_{g}$, converted into a logarithmic scale, and as a result, it returns the gesture, *g*, that has obtained the highest probability $P_{max}$. Additionally, a confidence index (CI) value between 0 (null confidence) and 1 (full confidence) is obtained. This index is computed by means of the two highest probabilities—that of the recognized gesture, $P_{max}$, and that of the gesture recognized in the second position, $P_{2}$ (9):

(9) $$\mathrm{CI}=min\left(\frac{P_{2}-P_{max}}{2P_{max}},\;1\right).$$

The dynamic gesture library is updated with the information of the record that has just been recognized through the transition (**U**) and emission probability matrices (**V**). Each time a new record, *E*, has to be recognized, the transition and emission probability matrixes of this new sequence are computed. They are used to obtain the new matrixes of the gesture set $\lambda_{g}$ (7) that was just recognized, using (10) and (11):

(10) $$U_{h}=\left(1-p\right)U_{h-1}+pU_{new},$$

(11) $$V_{h}=\left(1-p\right)V_{h-1}+pV_{new},$$

where $U_{h}$ is the new transition probability matrix of the gesture set $\lambda_{g}$; $U_{h-1}$ is the previous transition probability matrix of the gesture set $\lambda_{g}$, and $U_{new}$ is the transition probability matrix of the new record. In the same way, $V_{h}$ is the new emission probability matrix of the gesture set $\lambda_{g}$; $V_{h-1}$ is the previous one, and $V_{new}$ is the emission probability matrix of the new record. Finally, *p* is the value used to weight the new record with respect to the old ones. Depending on its value, the gesture library is updated using a different amount of information from the newly recognized record. High values of *p* make the gesture library include more information about the new record than of the previous ones. On the contrary, small values of *p* mean that the library is barely modified by the new information. Thus, this parameter is chosen so that the CI will be maximized, as explained next.

When the new record has been misrecognized, either because it is substantially different from the ones in the library, or because the surgeon has made a mistake, the surgeon informs the system through a voice command and the value of *p* is set to zero (i.e., this record is not used to update the dynamic gesture library). On the contrary, when the system properly recognizes the record, *p* must be chosen to improve the overall process. To avoid a static system where the weights, *p*, are defined and fixed during the design process, a reinforcement learning (RL) algorithm is used to make the system learn this parameter. This learning technique tries to maximize a reward that is received when the system makes a decision [24]. Within this work, the goal was to maximize the CI previously defined, so that the overall performance of the recognition process increases. Thus, the reward signal of the RL algorithm will be the confidence index as defined in (8). On the other hand, the decision to be taken is which weight value, *p*, will be used to update the dynamic gesture library. As this decision also depends on the CI value, the RL algorithm is organized to make independent decisions according to the level of confidence on the recognition. Thus, records with high CI will tend to higher weighting values than the ones recognized with extremely low CI. Although the CI can take values between 0 and 1, in order to avoid having a different weight value for each possible CI, three different ranges of the CI value are established. This way, only three weight values, *p*, are learned—one per CI group (i.e., CI = [0.0–0.5), [0.5–0.8), [0.8–1.0]). As can be observed, the size of each group decreases as the CI value increases (i.e., recognitions with CI ∈ [0.8–1.0] are more relevant than the ones in [0.5–0.8) and even more than the ones in [0.0–0.5)). This makes it so that only the best recognitions (higher CI) have a high impact on the dynamic gesture library update.

On the other hand, the weight values, *p*, have to be discretized in order to be used in the RL process (i.e., the decision has to be made within a finite set of predefined values. To make the process converge in a reasonable number of detections, it has to be reduced. Thus, it is discretized, taking the following considerations into account: (1) New recorded matrices cannot completely substitute the previous ones in the gesture set because all the historic information would be lost; thus p=1 is discarded. (2) New record matrixes cannot be rejected if the gesture has been correctly detected, thus p=0 is discarded. (3) The gesture set has to include plenty of information from several sources, that is, different gesture records. To achieve this, the values of *p* are preferred not to be too high because higher values imply the loss of information of the previous records. Consequently, the possible values of *p* are discretized into: p=0.2,0.3,0.4,0.5,0.6.

According with the discretization and aggrupation that has been made, the RL algorithm has different weight values to choose per CI group. That is, for each CI value within a group, the RL algorithm has five different *p* values to choose. This range of possibilities constitute the set of state–action pairs that defines this kind of algorithm and are implemented as any other production rule in the procedural memory. The states are the different CI groups and the action is the weight value selection (Table “Pairs” in Figure 7).

To be able to make the decision, that is, to choose a *p* value within the five possible ones within a CI group, these action–state pairs (CI group—*p*) are weighted. Each pair is associated to a *Q*-value that will be used by the RL algorithm to make the decision. The *Q*-value is updated when the reward is received, following (12) (SARSA algorithm):

(12) $$\delta_{h}=\alpha \left(r_{h+1}+\;\gamma Q_{h+1}-\;Q_{h}\right),\;\;\;$$

where $Q_{h+1}$ is the *Q*-value in the h+i th iteration; $r_{h+1}$ is the reward collected in this iteration (i.e., the CI); α is the learning rate; and γ is the discount rate. The learning rate determines the importance of new knowledge over old information, while the discount factor determines the importance of future rewards [32]. The last two parameters are considered as initial conditions of the RL algorithm and are set as preconditions in the experiments.

As the RL technique is based on discovering which actions are the most rewarded by trying them [24], this kind of algorithm is a trade-off between exploration and exploitation: the system has to exploit what it already knows, but it also has to explore new actions. To determine how the rules are selected based on their *Q*-value, the ϵ—greedy exploration strategy [33] is used. This strategy randomly selects a pair (CI group—*p*) with ϵ probability, while the pair with the highest *Q*-value is selected with 1−ϵ probability.

### 2.5. Co-Worker Robotic Scenario for HALS Kidney Resection

**Figure 8:**
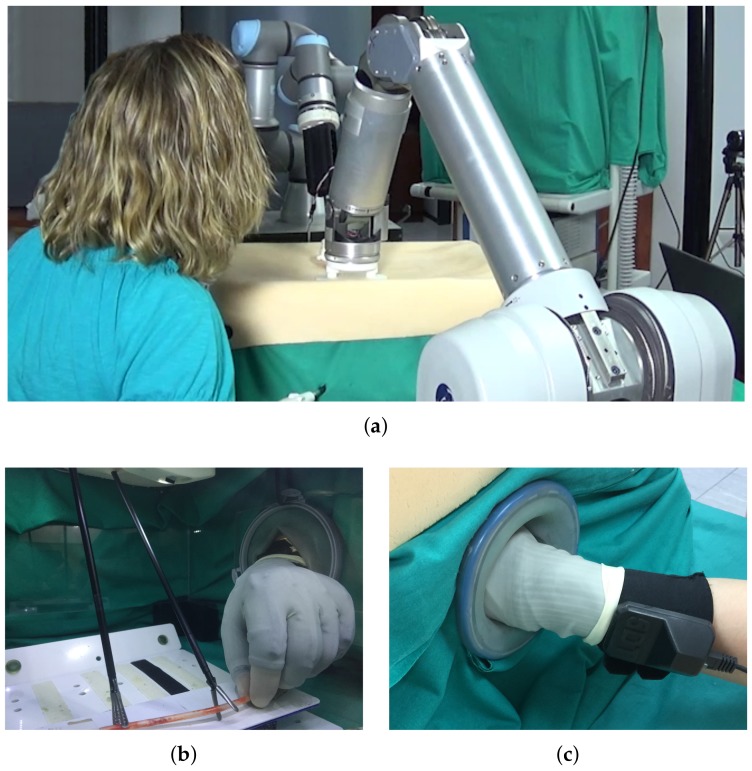
Experimental setup. (**a**) Co-worker robot assistant formed by two robotic arms that handle a surgical tool and a stereoscopic mini-camera. (**b**) The inside of the abdominal simulator. (**c**) Detail of the HandPort used to insert the hand into the abdominal simulator.

The HGR system proposed by this work was tested on a HALS protocol of a total kidney resection, where the ureter and blood vessels were clipped. The experimental setup, which is shown in Figure 8a, emulated a HALS co-worker robotic surgery scenario, using commercial devices. It included two robots: a WAM manipulator from Barret Technology that magnetically handled a stereoscopic mini-camera [26], and a UR3 manipulator from Universal Robots that controlled a surgical tool. The abdominal simulator dimensions were 37×47×20 cm ${}^{3}$, which is in the range of the commercial ones found in the market for abdominal laparoscopic training [34]. The surgeon managed one conventional surgical tool with one hand. The surgeon’s other hand ws introduced into the abdominal simulator through a HandPort [35], as shown in Figure 8b,c. This HandPort is a commercial tool from Endopath Dextrus that allows up to 4 cm abdominal wall thickness [36]. Moreover, the surgeon’s hand was equipped with a smart Data Glove Ultra from Fifth Dimension Technology. This glove gives the position of the fingertips as well as the flexion of the finger joints. The fingertips are open, so the surgeon can have the same tactile sensation as without the glove. Although this configuration has only been tested on in-vitro experiments, the glove could be protected with an extra surgical glove, so it can be isolated from the patient, which prevents a further sterilization of the device.

**Figure 9:**
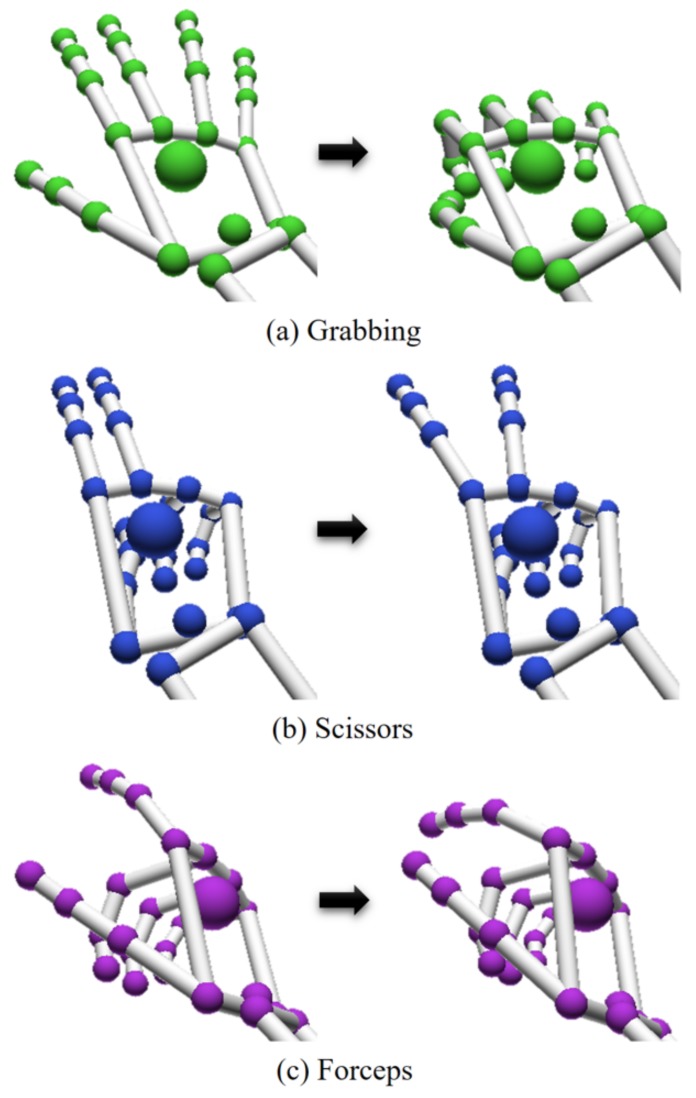
Set of surgical gestures needed for the kidney resection protocol for HALS.

The stages of the total kidney resection of the HALS co-worker scenario proposed in this paper are shown in Table 1. Although there is no limit to the number of gestures managed by the proposed algorithm, the kidney resection protocol only needs three different gestures. Each stage of this protocol can be described by the recognition of a set of surgical gestures made by the surgeon’s hand (Figure 9). The selection of these gestures considers that the surgeon is able to make their related finger displacement in a HALS in-vitro environment (Figure 8):

- Grabbing. This gesture consists of opening and closing the hand. It is useful to detect surgical situations like grabbing a big mass of tissue or an organ, although it can also be made for separating adhesions (stages 1 and 6).
- Scissors. With the index and middle fingers extended, the surgeon separates and joins their tips like a scissor mechanism. This can be used to order the robot tool to make an action with its tool (stage 4).
- Forceps. The thumb and index fingertips get closer and retreat like forceps. This may apply to detecting when the surgeon’s hand is pulling a thin tissue or vessel (stage 3).

As shown in Figure 3, the recognition of a new gesture makes the system progress to the next stage. Then, the gesture library is updated through the process explained in Section 2.4 and a new action is commanded to the endoscope and/or the robotized surgical tool, as described in Section 2.2.

This protocol and the gesture recognition process itself entails knowledge that has to be included into the semantic memory. Specifically, related to the protocol, the first semantic unit has to include the HALS protocol, so it is encoded as $S_{11}=<1,KidneyResection>$. The information about the protocol stages and the triggers that make the protocol to progress from one stage to the next is codified in the second semantic unit as:

(13) $$\begin{array}{cc} \; & S_{21}=<1,stage_{1},grabbing\;hand\;gesture>,\\ \; & S_{22}=<1,stage_{2},forceps\;insertion>,\\ \; & S_{23}=<1,stage_{3},forceps\;hand\;gesture>,\\ \; & S_{24}=<1,stage_{4},scissor\;hand\;gesture>,\\ \; & S_{25}=<1,stage_{5},scissor\;insertion>,\\ \; & S_{26}=<1,stage_{6},grabbing\;hand\;gesture>. \end{array}$$

Finally, the semantic unit $S_{3}$ stores the robot actions as follows:

(14) $$\begin{array}{cc} \; & S_{31}=<1,stage_{1},endoscope\;movement>,\\ \; & S_{32}=<1,stage_{2},forceps\;movement>,\\ \; & S_{33}=<1,stage_{2},endoscope\;movement>,\\ \; & S_{34}=<1,stage_{3},endoscope\;movement>,\\ \; & S_{35}=<1,stage_{4},endoscope\;movement>,\\ \; & S_{36}=<1,stage_{5},scissors\;movement>,\\ \; & S_{37}=<1,stage_{5},endoscope\;movement>,\\ \; & S_{38}=<1,stage_{6},endoscope\;movement>. \end{array}$$

Related to the gesture recognition process, in order for each type of gesture to be recognized, its set, $\lambda_{g}$, has to be included. Likewise, the discretization of the weight values and the aggrupation of the CI, needed for the on-line update of the dynamic gesture library, must also be included.

Three new semantic units are needed to codify this information, what makes the semantic memory finally have a total of six units.

Specifically, $S_{4}$ includes the gesture type and the parameters included in its set:

(15) $$S_{4j}:=<gesture_{j},SQ_{j},SE_{j},U_{j},V_{j},\pi_{j}>,$$

where for each gesture, SQ denotes the states of the HMM, SE is the set of all possible combinations of the encoded sequences, U is the transition matrix, V is the emission matrix, and π is the initial states distribution as defined in Section 2.4. For the HALS protocol defined in Table 1, this semantic unit will be encoded as:

(16) $$\begin{array}{cc} \; & S_{41}=<grabbing,SQ_{1},SE_{1},U_{1},V_{1},\pi_{1}>,\\ \; & S_{42}=<scissors,SQ_{2},SE_{2},U_{2},V_{2},\pi_{2}>,\\ \; & S_{43}=<forceps,SQ_{3},SE_{3},U_{3},V_{3},\pi_{3}>. \end{array}$$

$S_{5}$ includes the discretization of the weighting factor, *p*, of the on-line process:

(17) $$S_{5}=<0.2,0.3,0.4,0.5,0.6>.$$

Finally, $S_{6}$ contains the aggrupation of the CI.

(18) $$\begin{array}{c} S_{61}=<0.0,0.5>,\\ S_{62}=<0.5,0.8>,\\ S_{63}=<0.8,1.0>. \end{array}$$

On the other hand, the entire gesture recognition process and the dynamic gesture library update process form part of the procedural memory (Section 2.2.2). Thus, the whole HALS workflow shown in Figure 3 is encoded into the production rules ($PR_{l}$) shown in Table 2. The first one, $PR_{1}$, is devote to the recognition of a new gesture. When the perception system detects a new record, *E* obtained with (9), the gesture recognition process explained in Section 2.4 starts ($\left[g,\;U_{g},\;V_{g}\right]=recognition\left(E,S_{4}\right)$). Once a new gesture, *g*, has been recognized, two actions are made: ($PR_{2}$) the HALS protocol stage is updated ($new\_stage=\;update\_stage(g,\;S_{4})$) and ($PR_{3}$) the RL algorithm selects a new weight, p=RL_algorithm(CI), to later ($PR_{4}$) update the dynamic gesture library with the information of the recently recognized gesture $\left[U_{h},V_{h}\right]=library\_update\left(p,U_{h-1},V_{h-1},U_{\mathrm{new}},V_{\mathrm{new}}\right)$. When a new stage is detected, ($PR_{5}$) is performed by the surgical robot to properly assist the surgeon, executed by the action system ($execute\_actions(new_{s}tage,\;S_{3})$). Finally, if there is nothing new ($PR_{6}$) the cognitive system enters into a waiting state. Each of these six productions are codified as a larger number of rules.

## 3. Discussion and Results

The proposed experiments focused on the hand gesture recognition process detailed in this paper. In particular, the objective of the experiments was to compare the overall performance of the gesture recognition process detailed in this paper. The comparison was made among three different configurations of the algorithm: using only the gesture recognition process with no dynamic gesture library update (Only Recognition); using the gesture recognition process coupled with the on-line update of the dynamic gesture library explained in Section 2.4 but with fixed weight (p=0.5)—that is, no RL algorithm to infer the weight values (Recognition + *p* = 0.5); and finally, the whole process including the RL approach (Recognition + RL).

For the third configuration used in the experiments (i.e., the one that used the RL algorithm), the discount rate (γ) was set to 0.9 and the learning rate (α) to 0.3, making the system consider past information but also new information. Regarding the use of new knowledge to explore, the ϵ—greedy probability (ϵ) was configured with an initial value of 0.4 in the first 20 gestures to allow exploration of new weight values. Then, it was reduced to 0.2 to get a higher exploitation of the already known values and thus enable the convergence of the weight factor.

**Figure 10:**
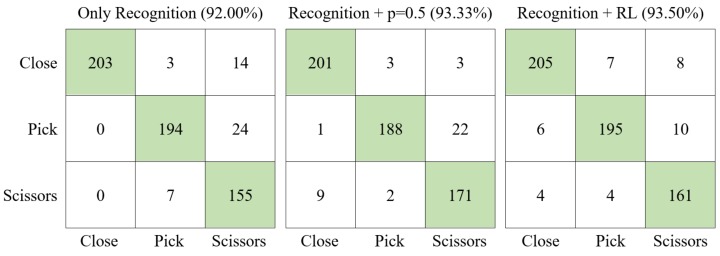
Confusion matrices for each configuration of the recognition algorithm.

The offline training process introduced in Section 2.3 was performed with 30 repetitions of each hand gesture explained above (Figure 9). The topology of the HMMs associated to each gesture was the same as described in Section 2.3.2. The obtained dynamic gesture library was used to make realizations of the three configurations already explained by ten different users. Each realization included the detection of 60 gestures. The results obtained by each configuration of the algorithm described above are shown in the form of confusion matrices in Figure 10. Each row represents the performed gesture, and each column represents the detected gesture. So, the diagonal (green) represents the gestures that were correctly detected. The rows and columns sum different values because each user did each gesture different times in a random order. This method prevents the user from making very similar gestures because of the continuous repetitions. Thus, the only condition consists of getting a total of 60 repetitions per user so the total sum of all elements of each confusion matrix is 600. From the results obtained, it can be noticed that the percentage of correct gesture detection was slightly higher when the RL algorithm is used. The obtained values were equivalent to the ones analyzed in [13].

**Figure 11:**
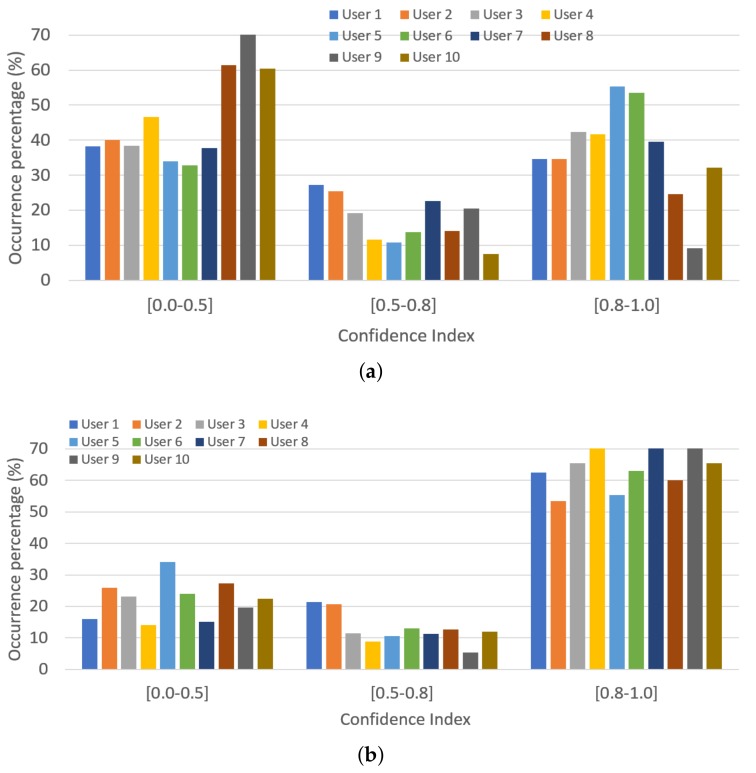
Occurrence percentage of well-detected gestures depending on their confidence index. (**a**) Without the on-line learning algorithm; (**b**) With the on-line RL algorithm.

Coupled with the type of gesture detected, the process produces the confidence index of the well-detected gesture, which informs about the reliability of the detection. The closer the confidence index is to 1, the more reliable the detection. The CI results for all the realizations made by all subjects are shown in Figure 11. A change can be seen in the tendency of the CI. When no RL algorithm was used, the most populated range of well-detected gestures was the one with CI in the [0.0–0.5) range; however, when the RL process was used, the bigger range was the opposite one, [0.8–1.0]. The mean of well-detected gestures with CI in [0.8–1.0] for all users changed from 37.08% when no RL algorithm was used to 65.51% when it was used.

A deeper analysis of the experiment results shows that the number of well-detected recognitions with full confidence (CI =1.0) in the results also increased when the RL algorithm was used (Table 3). This result coupled with the previous one made it so the overall false positives were reduced when the RL algorithm was used. The previous results reveal that the contribution of the on-line RL process not only increased the total percentage of well-detected gestures, but it also improved the performance of the recognition process, as its confidence index increased as well.

**Figure 12:**
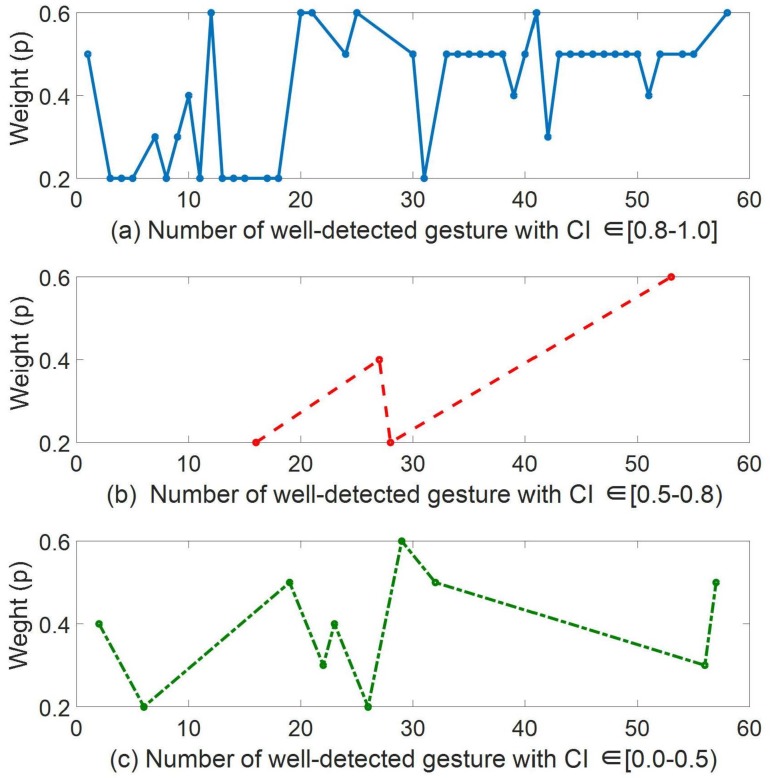
Weight value (*p*) learning of Realization 1 of User 1. The learning was divided into the three aggrupations of the confidence index.

When the configuration with the on-line RL process was used, three different weight factors were learned, as explained in Section 2.4. A weight factor was learned for detections made with a confidence index in the [0.0–0.5) range, another one for detections where the CI was within [0.5–0.8); and finally, the last weight factor for detections with CI in [0.8–1.0]. Figure 12 shows the evolution of these three weight factor values, *p*, in Realization 1 of User 1, when RL was used. The remaining ones behaved in the same way.

As can be seen in Figure 12a (CI ∈ [0.8–1.0]), at the beginning of the learning process there was a high variability in the weight value, but because of the reward feedback it finished converging to p=0.5. This value is one of the upper possible ones, which makes the contribution of the new gesture to the library high when the confidence index is also high. For the well-detected gestures with a lower CI, there were not enough samples to make the algorithm converge, as shown in Figure 12b,c. That is, the number of well-detected gestures with CI lower than 0.6 reduced significantly when the RL algorithm was used.

Through these results it can be stated that the use of the RL on-line process improved the number of well-detected gestures and the overall performance of the recognition process. Although the accuracy of the system was not heavily improved when the RL process was used (Figure 10), the confidence in the well-detected recognitions was increased over 20% in relation to the use of only the Recognition process. The percentage of recognized gestures increased by switching from low to high confidence index, making the system more reliable.

Furthermore, in order to test the real-time capabilities of the algorithm, the mean response time was measured. On average, the algorithm spent 0.065 s in recognizing a gesture. As the time required to record the gesture is about 2 s on average—greater than the one needed to the recognition process—the real-time capabilities are assured as stated in [37]. On the other hand, a user feels that a system is reacting instantaneously if the time response is lower than 0.1 s [38], which is met in the recognition process of the proposed algorithm.

Finally, an additional experiment was considered to validate the choice of the HMM network as the core process of the proposed algorithm. Toward this end, User 2 made 30 repetitions of each of the three gestures shown in Figure 9 for training both a conditional random field (CRF) and an HMM algorithm. Then, this user performed three realizations, each of them consisting of the detection of 60 gestures on a random sequence. Again, the same data acquired were used in parallel by the CRF and the HMM. The average percentage of correctly detected gestures was 88.33% for the CRF and 89.44% for the HMM.

In this way, the CI behavior was also analyzed. As seen in Table 4, the CIs when the gesture was correctly detected were higher when the CRF algorithm was used. However, when the CRF gave a false positive (i.e., when the gesture was not correctly detected), its confidence index was much higher than the one obtained in HMM.

**Figure 13:**
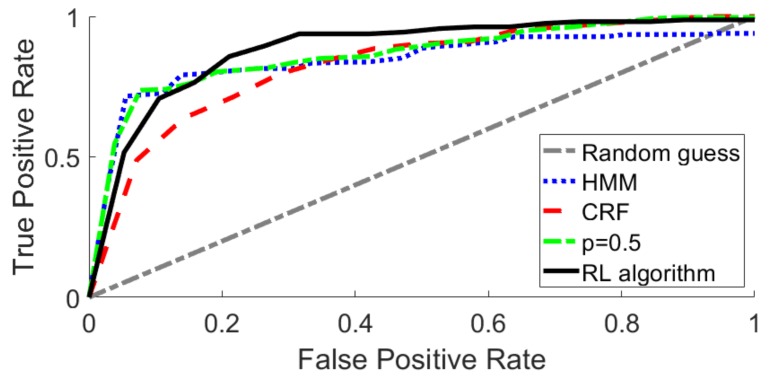
Receiver operating characteristic (ROC) curves for each of the recognition algorithms analyzed in the experiments: HMM, CRF, update training with constant p=0.5, and RL.

The experimental results obtained for each recognition methodology were compiled in the form of a receiver operating characteristic (ROC) curve. Figure 13 shows the ROC curves of all the experiments with the true positive rate (TPR) versus the false positive rate (FPR) data. The accuracy parameter is the area under each of the curves, and quantifies the performance of the recognition algorithm. The static HMM algorithm with no update of the gesture library gave an accuracy of 0.8502, whereas the CRF algorithm had about the same value (0.8436). The update of the HMM gesture library with a constant gain of p=0.5 improved the accuracy up to 0.8720. However, the RL algorithm gave the best results, with an accuracy of 0.9085.

## 4. Conclusions

The experimental results of this HGR system demonstrated how the on-line reinforcement learning process made the system improve its overall performance, increasing the confidence of each recognized gesture. Additionally, the proposed approach was compared with a CRF algorithm. Although the recognition rates were quite similar, the confidence index of false positives in CRF was higher than with the HMM. Thus, the use of the HMM model, improved with the reinforcement learning algorithm, increased the mean confidence index, suggesting it is a more suitable approach for surgical applications. The obtained results agree with the values obtained from other works with different methodologies, such as recurring neural networks (91.9%) [13] or dynamic time warping (91.4%) [17]. However, it can be remarked that the use of the RL algorithm may slightly improve both the accuracy and the CI, as was shown in the experiments.

However, this approach presents some limitations that should be solved. One of these is the surgeon-dependency—that is, the gestures that are trained can only be detected with the same surgeon that performed the training. The use of a cognitive architecture would enhance the use of a modified algorithm to combine the information of all the training into a single common gesture library. In this way, any surgeon could benefit from the previous work and collaborate with new data thanks to the online recognition process. On the other hand, when the recognition system fails, the feedback is provided by voice commands, which would increase the surgeon’s workload. In this sense, the success rate could be increased by means of a multi-sensor system, which would take into consideration other sensors such as a vision system and/or the MYO armband. Finally, during the experimental stage, only three gestures were trained and evaluated. Although these gestures were considered sufficient in the proposed surgical scenario, they could be extended to evaluate the proposed approach performance as the number of trained gestures are increased.

Regarding future research directions, our aim is to integrate the proposed gesture recognition approach as a subsystem within the architecture shown in Figure 2. Thus, a complete cognitive system would be developed, integrated, and tested in a real surgical scenario.

## Figures and Tables

**Table 1:** Kidney resection protocol for HALS.

Stage	Tool	Gesture	Robot Action
1	Hand	*Grabbing Hand Gest.*	Endoscope Motion
2	Forceps	Forcep Insertion	Forcep and Endoscope Motion
3	Hand	*Forcep Hand Gest.*	Endoscope Motion
4	Clipper	*Scissor Hand Gest.*	Endoscope Motion
5	Scissor	Scissor Insertion	Scissor and Endoscope Motion
6	Hand	*Grabbing Hand Gest.*	Endoscope Motion

**Table 2:** Production rules.

$PR_{1}:\mathrm{IF}\;E\;\mathrm{THEN}\;[g,U_{g},V_{g}]=recognition(E,S_{4})$
$PR_{2}:\mathrm{IF}\;g\;\mathrm{THEN}\;new\_stage=\;update\_stage\left(g,S_{4}\right)$
$PR_{3}:\mathrm{IF}\;g\;\mathrm{THEN}\;p=RL\_algorithm\left(CI\right)$
$PR_{4}:\mathrm{IF}\;p\;\mathrm{THEN}\;\left[U_{h},V_{h}\right]=library\_update\left(p,U_{h-1},V_{h-1},U_{\mathrm{new}},V_{\mathrm{new}}\right)$
$PR_{5}:\mathrm{IF}\;new\_stage\;\mathrm{THEN}\;execute\_actions\left(new_{s}tage,\;S_{3}\right)$
$PR_{6}:\mathrm{IF}\;nothing\;\mathrm{THEN}\;wait$

**Table 3:** Well-detected gestures with CI =1.0.

	Only Recognition	Recognition + *p* = 0.5	Recognition + RL
User 1	14.30%	47.17%	60.55%
User 2	14.72%	38.83%	41.95%
User 3	31.63%	43.66%	56.33%
User 4	24.44%	40%	62.32%
User 5	36.67%	45%	59.62%
User 6	24.44%	39%	49%
User 7	16.85%	36.66%	48.33%
User 8	19.76%	46.66%	60%
User 9	20.27%	45%	60%
User 10	26.08%	38.33%	43.33%

**Table 4:** Average confidence index. CRF: conditional random field; HMM: hidden Markov model.

	Misrecognized Gesture		Well-Detected Gesture	
	HMM	CRF	HMM	CRF
Experiment 1	0.3161	0.4137	0.6984	0.8681
Experiment 2	0.1843	0.4540	0.6998	0.8166
Experiment 3	0.1455	0.5280	0.6696	0.8449

## Abbreviations

The following abbreviations are used in this manuscript:

MIS	Minimally Invasive Surgery
HALS	Hand-Assisted Laparoscopic Surgery
HMM	Hidden Markov Model
SILS	Single-Incision Laparoscopic Surgery
HMI	Human–Machine Interface
HGR	Hand Gesture Recognition
CRF	Conditional Random Field
DTW	Dynamic Time Warping
GMM	Gaussian Mixture Model
RL	Reinforcement Learning
CI	Confidence Index

## References

[1] Zorn L., Nageotte F., Zanne P., Legner A., Dallemagne B., Marescaux J., de Mathelin M. (2018). A Novel Telemanipulated Robotic Assistant for Surgical Endoscopy: Preclinical Application to ESD. IEEE Trans. Biomed. Eng..

[2] Fujii K., Gras G., Salerno A., Yang G.Z. (2018). Gaze gesture based human robot interaction for laparoscopic surgery. Med. Image Anal..

[3] Zhou T., Pablo Wachs J. (2018). Early prediction for physical human robot collaboration in the operating room. Auton. Robot..

[4] DiPietro R., Lea C., Malpani A., Ahmidi N., Vedula S.S., Lee G.I., Lee M.R., Hager G.D. (2016). Recognizing Surgical Activities with Recurrent Neural Networks. Medical Image Computing and Computer-Assisted Intervention.

[5] Bauzano E., Estebanez B., Garcia-Morales I., Munoz V.F. (2016). Collaborative Human–Robot System for HALS Suture Procedures. IEEE Syst. J..

[6] Ahmidi N., Tao L., Sefati S., Gao Y., Lea C., Haro B.B., Zappella L., Khudanpur S., Vidal R., Hager G.D. (2017). A Dataset and Benchmarks for Segmentation and Recognition of Gestures in Robotic Surgery. IEEE Trans. Biomed. Eng..

[7] Jacob M., Li Y.T., Akingba G., Wachs J.P. (2012). Gestonurse: A robotic surgical nurse for handling surgical instruments in the operating room. J. Robot. Surg..

[8] Jacob M.G., Li Y.T., Akingba G.A., Wachs J.P. (2013). Collaboration with a Robotic Scrub Nurse. Commun. ACM.

[9] Le H.T., Pham H.T.T. (2017). Hand Signal Recognition for Handling Surgical Instruments. Proceedings of the International Conference on the Development of Biomedical Engineering in Vietnam, Ho Chi Minh, Vietnam, 27–29 June 2017.

[10] Negin F., Rodriguez P., Koperski M., Kerboua A., Gonzàlez J., Bourgeois J., Chapoulie E., Robert P., Bremond F. (2018). PRAXIS: Towards automatic cognitive assessment using gesture recognition. Expert Syst. Appl..

[11] D’Orazio T., Marani R., Renò V., Cicirelli G. (2016). Recent trends in gesture recognition: How depth data has improved classical approaches. Image Vis. Comput..

[12] Xie R., Sun X., Xia X., Cao J. (2015). Similarity Matching-Based Extensible Hand Gesture Recognition. IEEE Sens. J..

[13] Pisharady P.K., Saerbeck M. (2015). Recent methods and databases in vision-based hand gesture recognition: A review. Comput. Vis. Image Underst..

[14] Galka J., Masior M., Zaborski M., Barczewska K. (2016). Inertial Motion Sensing Glove for Sign Language Gesture Acquisition and Recognition. IEEE Sens. J..

[15] Rossi M., Benatti S., Farella E., Benini L. Hybrid EMG classifier based on HMM and SVM for hand gesture recognition in prosthetics. Proceedings of the 2015 IEEE International Conference on Industrial Technology (ICIT).

[16] Tao L., Zappella L., Hager G.D., Vidal R. (2013). Surgical Gesture Segmentation and Recognition. Proceedings of the International Conference on Medical Image Computing and Computer-Assisted Intervention, Nagoya, Japan, 22–26 September 2013.

[17] Plouffe G., Cretu A.M. (2016). Static and Dynamic Hand Gesture Recognition in Depth Data Using Dynamic Time Warping. IEEE Trans. Instrum. Meas..

[18] Bautista M.A., Hernandez-Vela A., Escalera S., Igual L., Pujol O., Moya J., Violant V., Anguera M.T. (2016). A Gesture Recognition System for Detecting Behavioral Patterns of ADHD. IEEE Trans. Cybern..

[19] Raheja J., Minhas M., Prashanth D., Shah T., Chaudhary A. (2015). Robust gesture recognition using Kinect: A comparison between DTW and HMM. Optik.

[20] Duong N.H., Dang Hai H. A semi-supervised model for network traffic anomaly detection. Proceedings of the 2015 17th International Conference on Advanced Communication Technology (ICACT).

[21] Lima M., Zarpelão B., Sampaio L., Rodrigues J., Abrão T., Proença L. Anomaly detection using baseline and K-means clustering. Proceedings of the SoftCOM 2010: International Conference on Software, Telecommunications and Computer Networks.

[22] Song Y., Gu Y., Wang P., Liu Y., Li A. A Kinect based gesture recognition algorithm using GMM and HMM. Proceedings of the 2013 6th International Conference on Biomedical Engineering and Informatics.

[23] Ng A.Y., Jordan M.I. On Discriminative vs. Generative Classifiers: A comparison of logistic regression and naive Bayes. In Proceedings of the 14th International Conference on Neural Information Processing Systems.

[24] Sutton R., Barto A. (2018). Reinforcement Learning: An Introduction.

[25] Mishra R.K. (2009). Laparoscopic Tissue Approximation Techniques. Textb. Pract. Laparosc. Surg..

[26] Rivas-Blanco I., Lopez-Casado C., Perez-del Pulgar C.J., Garcia-Vacas F., Fraile J.C., Munoz V.F. (2018). Smart Cable-Driven Camera Robotic Assistant. IEEE Trans. Hum. Mach. Syst..

[27] Wang W., Tan A.H., Teow L.N. (2017). Semantic Memory Modeling and Memory Interaction in Learning Agents. IEEE Trans. Syst. Man Cybern. Syst..

[28] Muller M. (2007). Information Retrieval for Music and Motion.

[29] Pelleg D., Pelleg D., Moore A. X-means: Extending K-means with Efficient Estimation of the Number of Clusters. Proceedings of the 17th International Conference on Machine Learning.

[30] Hastie T., Tibshirani R., Friedman J. (2009). The Elements of Statistical Learning: Data Mining, Inference, and Prediction.

[31] Rabiner L. (1989). A tutorial on hidden Markov models and selected applications in speech recognition. Proc. IEEE.

[32] Laird J. (2012). The Soar Cognitive Architecture.

[33] Tokic M., Palm G. (2011). Value-Difference Based Exploration: Adaptive Control between Epsilon-Greedy and Softmax. KI 2011: Advances in Artificial Intelligence.

[34] EndoSurgical Laparoscopic Surgery Simulator. https://www.gtsimulators.com/EVE-Laparoscopic-Simulator-2nd-Generation-p/etx-a2-lap.htm.

[35] Goel A. (2015). HandPort Laparoscopic Surgery-Review and Current Status. Indian J. Surg..

[36] Endopath® Dextrus HALS—Euro-Medical. http://www.eu-medical.pl/produkty/dostep/chirurgia-laparoskopowa-z-asysta-reki-.

[37] Kuo S.M., Lee B.H. (2001). Real Time Digital Signal Processing.

[38] Nielsen J. (1993). Usability Engineering.

